# Supplementary material for: Eicosapentaenoic Acid Modulates Transient Receptor Potential V1 Expression in Specific Brain Areas in a Mouse Fibromyalgia Pain Model
Source: Int J Mol Sci. 2024 Mar 1;25(5):2901. doi: 10.3390/ijms25052901 (PMC10932372; doi:10.3390/ijms25052901)
Supplement: Supplementary file 1 [file ijms-25-02901-s001.zip › Supplementary figures.pptx]

## Slide 1
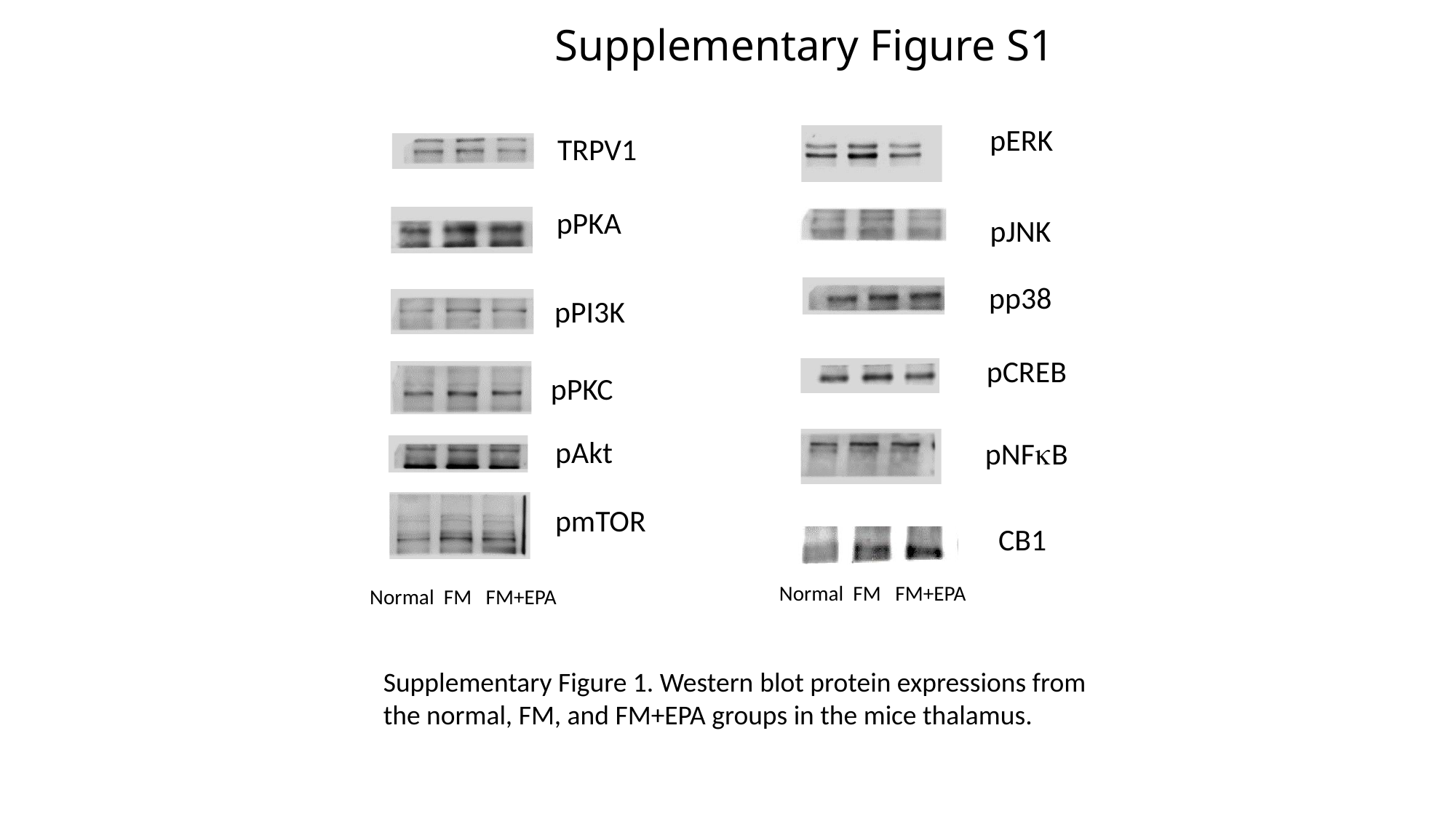

# Supplementary Figure S1
pERK
TRPV1
pPKA
pJNK
pp38
pPI3K
pCREB
pPKC
pAkt
pNFκB
pmTOR
CB1
Normal FM FM+EPA
Normal FM FM+EPA
Supplementary Figure 1. Western blot protein expressions from the normal, FM, and FM+EPA groups in the mice thalamus.

## Slide 2
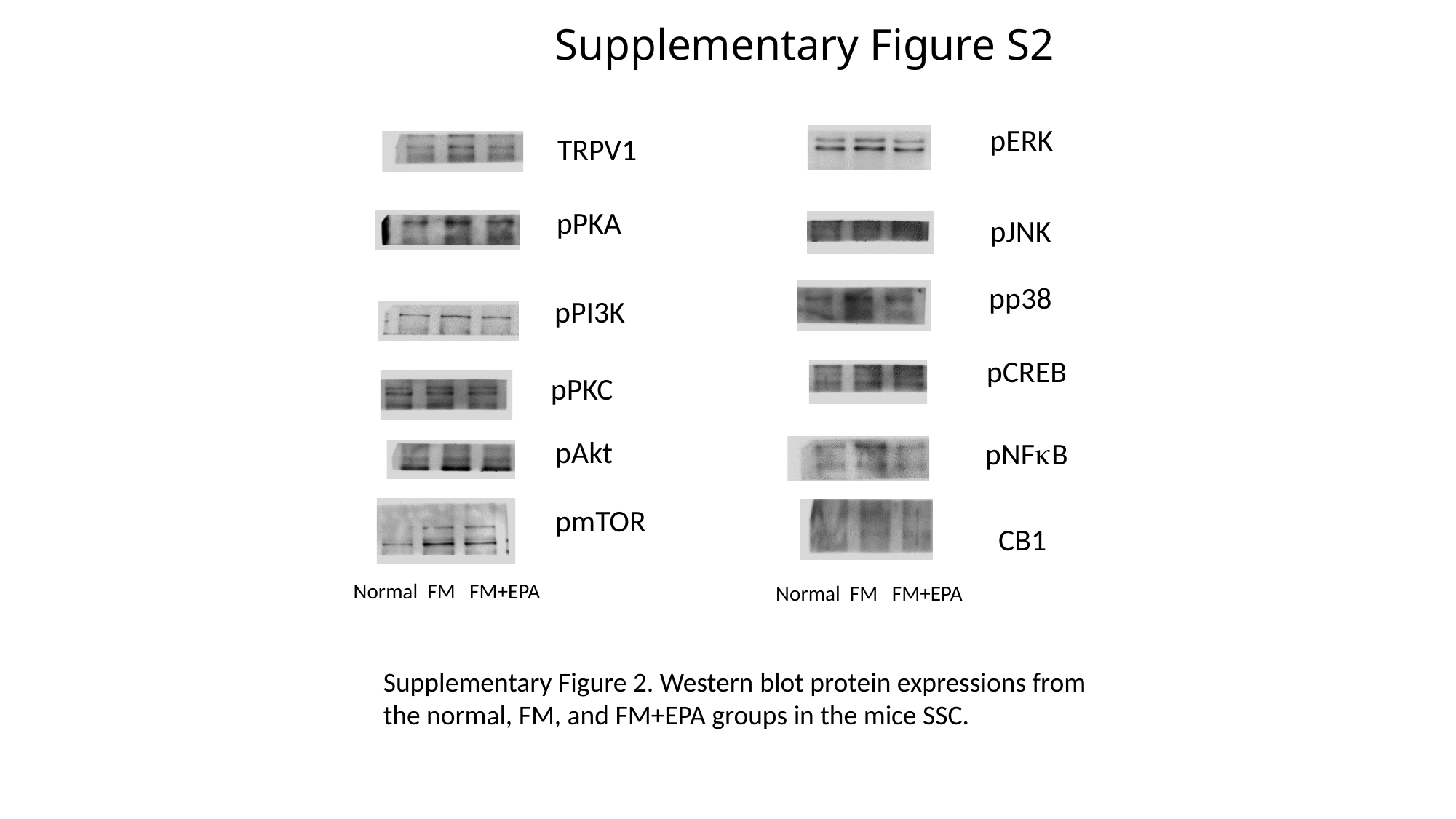

Supplementary Figure S2
pERK
TRPV1
pPKA
pJNK
pp38
pPI3K
pCREB
pPKC
pAkt
pNFκB
pmTOR
CB1
Normal FM FM+EPA
Normal FM FM+EPA
Supplementary Figure 2. Western blot protein expressions from the normal, FM, and FM+EPA groups in the mice SSC.

## Slide 3
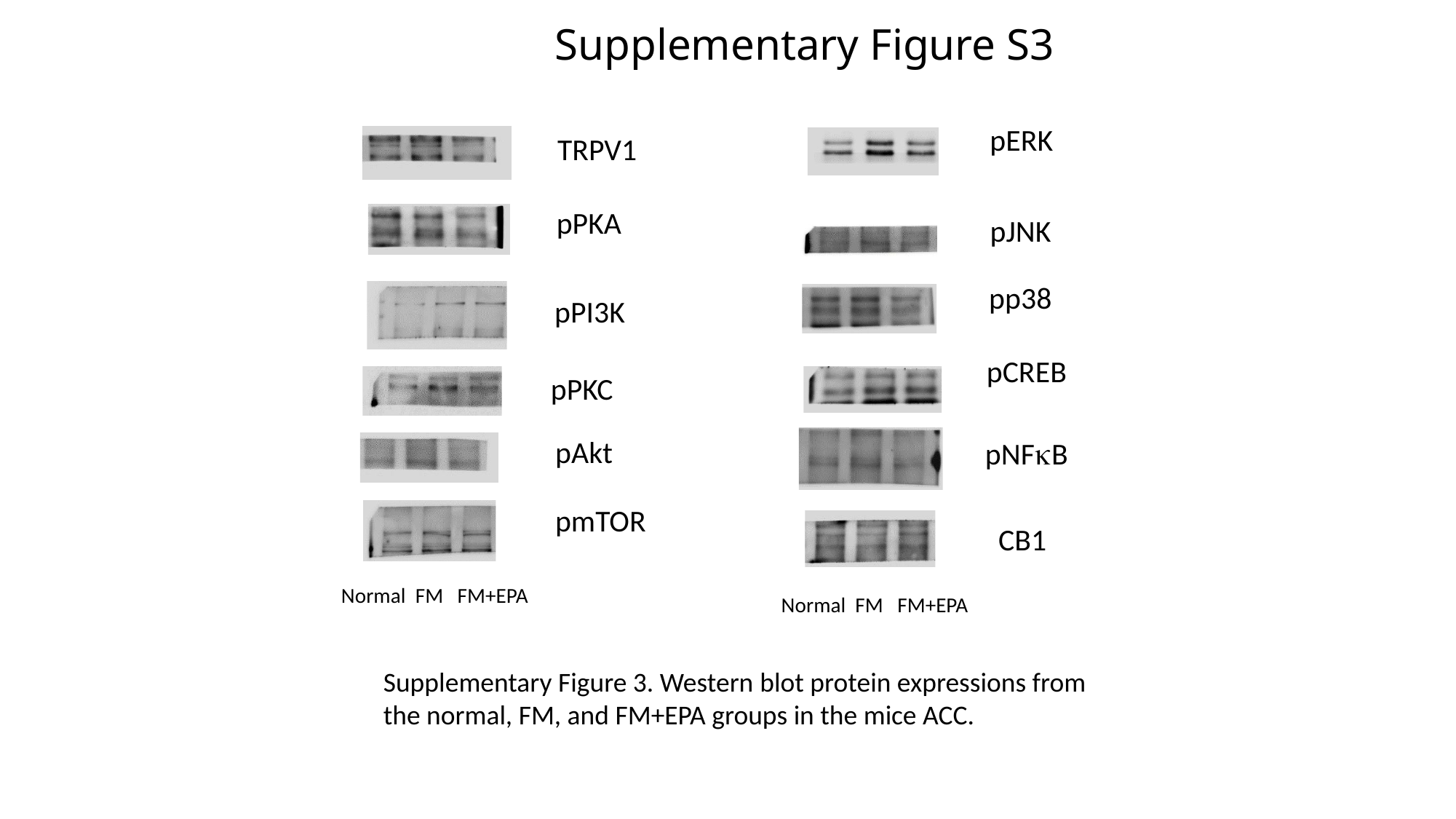

Supplementary Figure S3
pERK
TRPV1
pPKA
pJNK
pp38
pPI3K
pCREB
pPKC
pAkt
pNFκB
pmTOR
CB1
Normal FM FM+EPA
Normal FM FM+EPA
Supplementary Figure 3. Western blot protein expressions from the normal, FM, and FM+EPA groups in the mice ACC.

## Slide 4
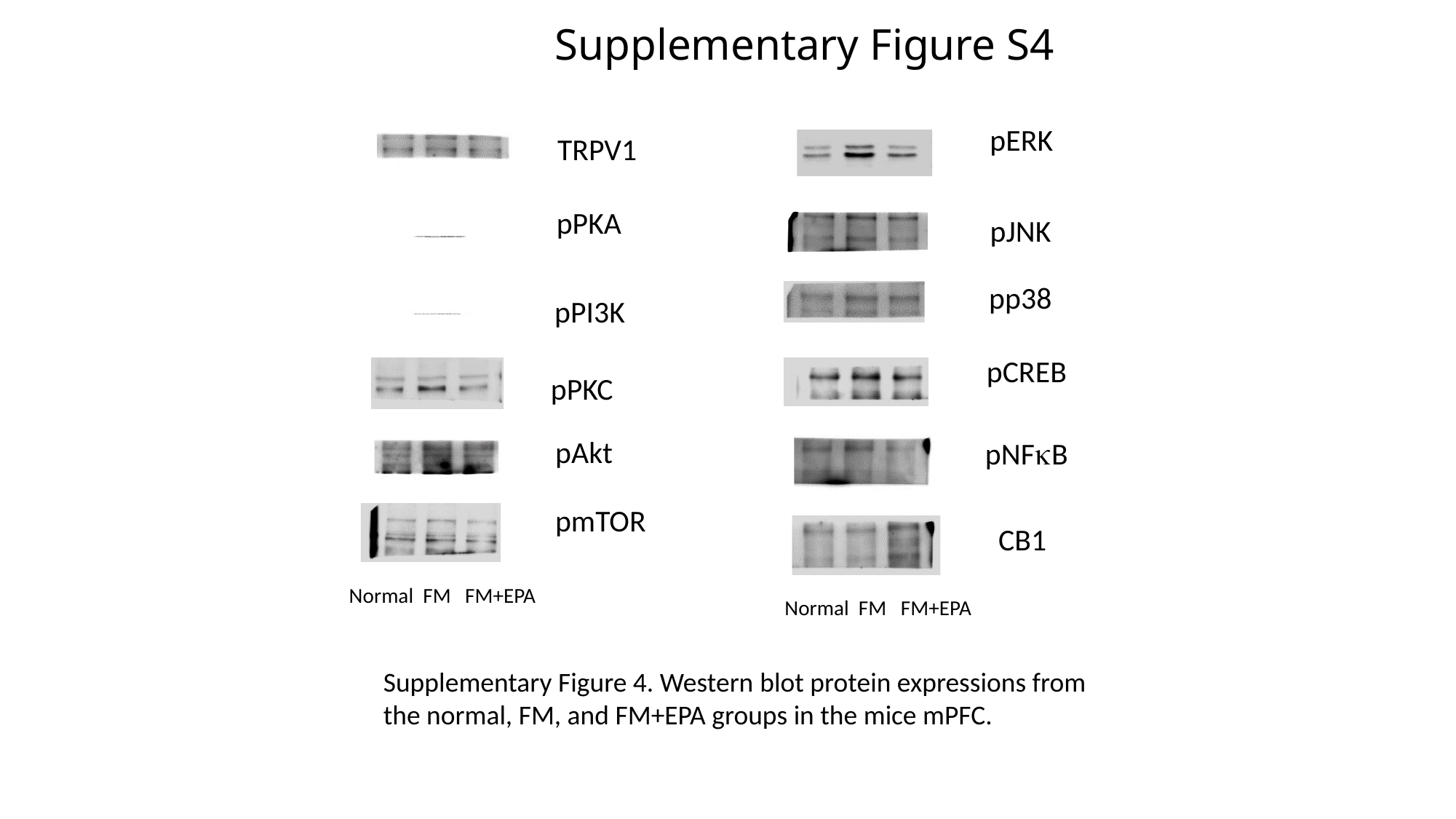

Supplementary Figure S4
pERK
TRPV1
pPKA
pJNK
pp38
pPI3K
pCREB
pPKC
pAkt
pNFκB
pmTOR
CB1
Normal FM FM+EPA
Normal FM FM+EPA
Supplementary Figure 4. Western blot protein expressions from the normal, FM, and FM+EPA groups in the mice mPFC.

## Slide 5
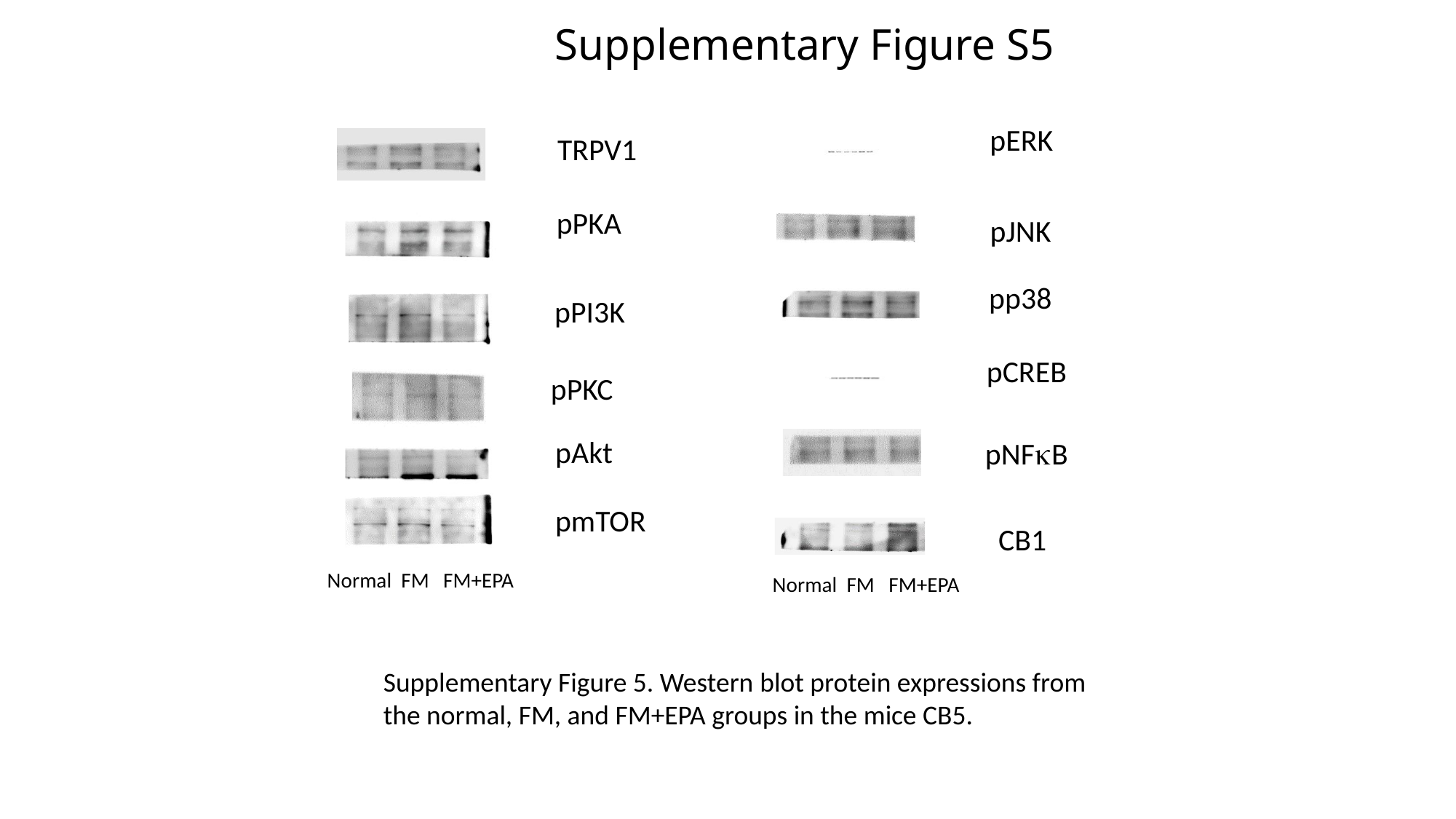

Supplementary Figure S5
pERK
TRPV1
pPKA
pJNK
pp38
pPI3K
pCREB
pPKC
pAkt
pNFκB
pmTOR
CB1
Normal FM FM+EPA
Normal FM FM+EPA
Supplementary Figure 5. Western blot protein expressions from the normal, FM, and FM+EPA groups in the mice CB5.

## Slide 6
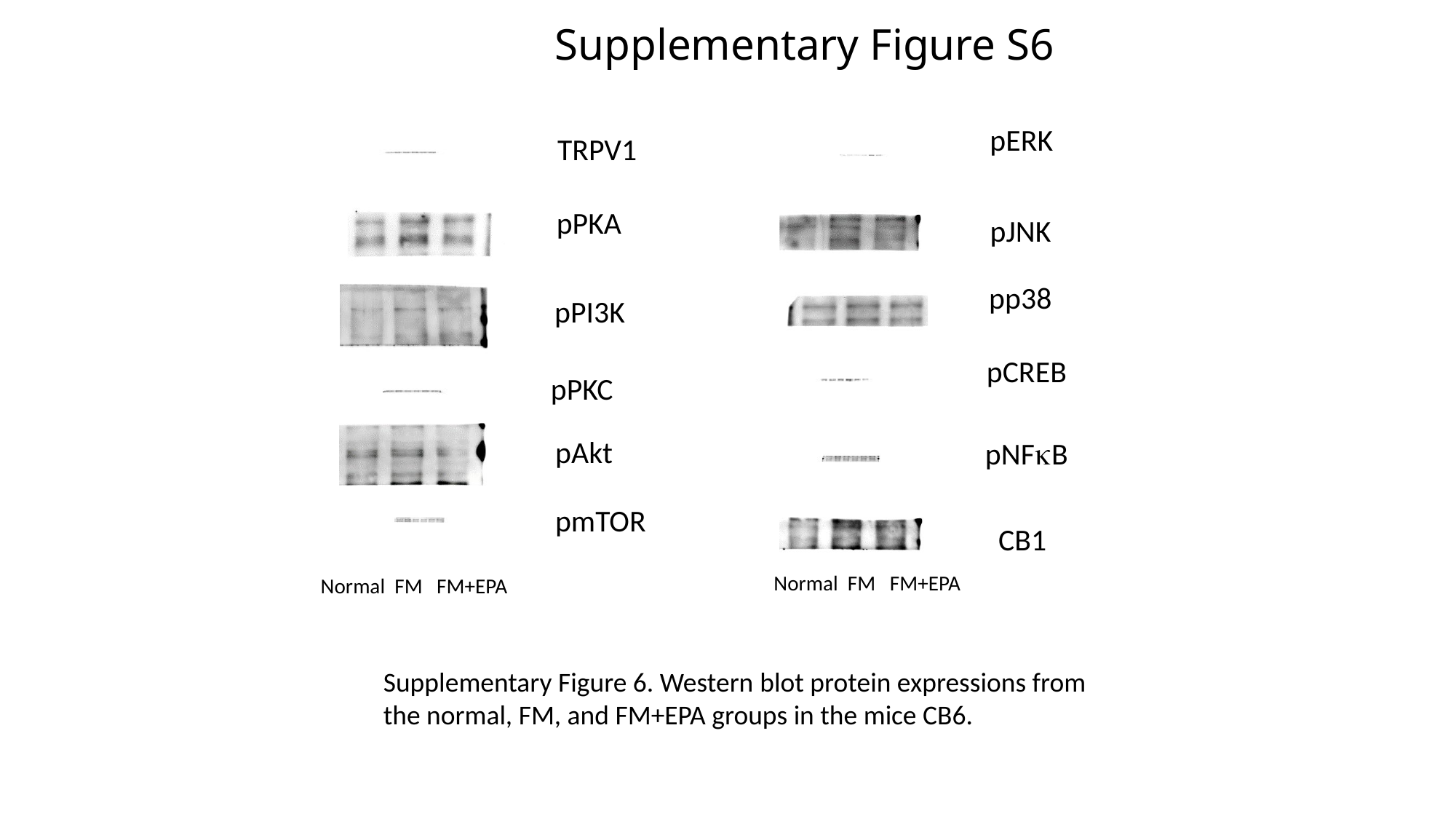

Supplementary Figure S6
pERK
TRPV1
pPKA
pJNK
pp38
pPI3K
pCREB
pPKC
pAkt
pNFκB
pmTOR
CB1
Normal FM FM+EPA
Normal FM FM+EPA
Supplementary Figure 6. Western blot protein expressions from the normal, FM, and FM+EPA groups in the mice CB6.

## Slide 7
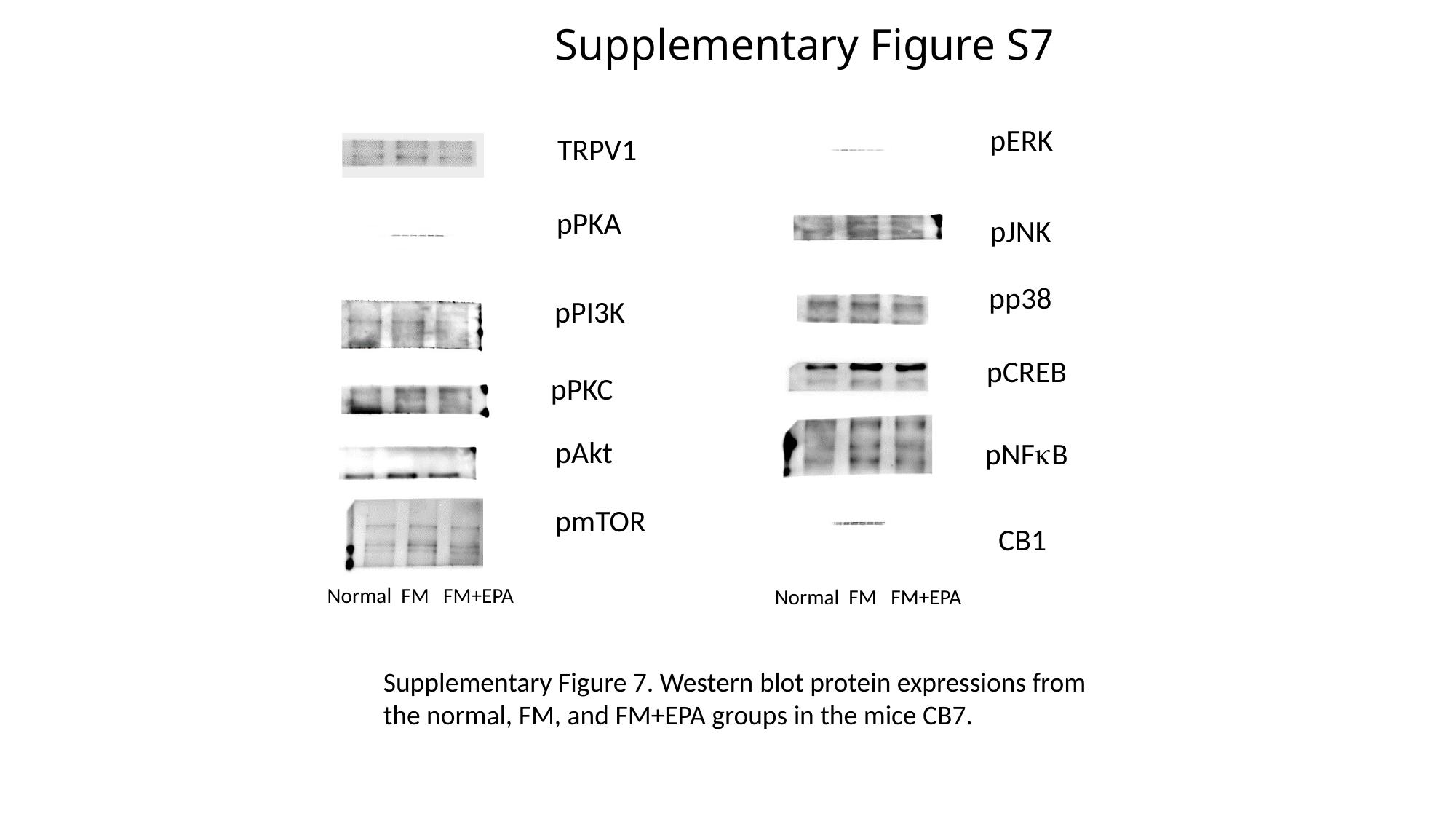

Supplementary Figure S7
pERK
TRPV1
pPKA
pJNK
pp38
pPI3K
pCREB
pPKC
pAkt
pNFκB
pmTOR
CB1
Normal FM FM+EPA
Normal FM FM+EPA
Supplementary Figure 7. Western blot protein expressions from the normal, FM, and FM+EPA groups in the mice CB7.
