# Supplementary material for: Eicosapentaenoic Acid Modulates Transient Receptor Potential V1 Expression in Specific Brain Areas in a Mouse Fibromyalgia Pain Model
Source: Int J Mol Sci. 2024 Mar 1;25(5):2901. doi: 10.3390/ijms25052901 (PMC10932372; doi:10.3390/ijms25052901)
Supplement: Supplementary file 1 [file ijms-25-02901-s001.zip › Supplementary table S1.docx]

| Primary Ab | Secondary Ab | Manufacturer |
| --- | --- | --- |
| TRPV1 | Goat Anti-Rabbit | Alomone, Israel, cat. no. ACC‑ 030. |
| pPKA | Goat Anti-Mouse | Santa CruzUSA, cat. no. SC‑ 12905. |
| pPI3K | Goat Anti-Rabbit | Novus, USA, cat. no. NBP2‑15071. |
| pPKC | Goat Anti-Rabbit | Millipore, USA, cat. no. 06-991. |
| pAkt | Goat Anti-Rabbit | Millipore, USA, cat. no. 05-802R. |
| pmTOR | Goat Anti-Rabbit | Millipore, USA, cat. no. 09-343. |
| pERK | Goat Anti-Rabbit | Millipore, USA, cat. no. 05-797R. |
| pJNK | Goat Anti-Rabbit | Millipore, USA, cat. no. 07-175. |
| pp38 | Goat Anti-Rabbit | Millipore, USA, cat. no. 09-272. |
| pCREB | Goat Anti-Rabbit | Millipore, USA, cat. no. 05-667. |
| pNF-κB | Goat Anti-Rabbit | Millipore, USA, cat. no. 06-418. |
| CB1 | Goat Anti-Rabbit | Millipore, USA, cat. no. AB9415. |
| α-tubulin | Goat Anti-Mouse | Novus, USA, cat. no. NB100-690. |
|  | Alexa Fluor™488-conjugated AffiniPure donkey anti-rabbit IgG (H+ L) | ThermoFisher, USA, cat. no. 21206. |
|  | Alexa Fluor™594-conjugated AffiniPure donkey anti-mouse IgG (H + L). | ThermoFisher, USA, cat. no. A-21203. |

Supplementary table 1. Primary-secondary antibodies and the manufacturers in the current study.
